# Managing periprosthetic joint infection—a qualitative analysis of nursing staffs’ experiences

**DOI:** 10.1186/s12912-022-00978-z

**Published:** 2022-07-18

**Authors:** Nike Walter, Bravena Wimalan, Susanne Baertl, Siegmund Lang, Thilo Hinterberger, Volker Alt, Markus Rupp

**Affiliations:** 1 Department for Trauma Surgery, University Medical Center Regensburg, Franz-Josef-Strauß-Allee 11, 93053 Regensburg, Germany; 2 Department for Psychosomatic Medicine, University Medical Center Regensburg, Franz-Josef-Strauß-Allee 11, 93053 Regensburg, Germany

**Keywords:** Perirosthetic joint infection, Qualitative approach, Nursing

## Abstract

**Background:**

Periprosthetic joint infection represents a major complication in orthopaedics and trauma surgery. For an ideal management approach, it is important to understand the distinct challenges for all persons involved in the treatment. Therefore, it was aimed at investigating (1) the impact of periprosthetic joint infection (PJI) on the well-being of nursing staff to (2) identify challenges, which could be improved facilitating the management of PJI.

**Methods:**

This is a qualitative interview study. In total, 20 nurses of a German university orthopedic trauma center specialized on infectious complications were recruited using a purposive sampling strategy. Content analysis was performed on transcripts of individual in-person interviews conducted between March 2021 and June 2021.

**Results:**

Three major themes could be extracted including (i) feelings associated with the management of PJI and the need for emotional support, illustrating the negative emotional impact on nurses, whereby receiving collegial support was perceived as an important coping strategy, (ii) patients’ psychological burden, highlighting the nurses’ lack of time to address mental issues adequately and, (iii) realization of the severity of PJI and compliance problems.

**Conclusion:**

Identified facilitating factors for PJI management include strengthening of mental care in the treatment of PJI, providing opportunities for exchange among multidisciplinary team members and implementing compliance-enhancing strategies. The findings of this study can be beneficial for improving professionals’ satisfaction, optimising the work environment, creating organizational structures which enhance opportunities for exchange and preventing mental health issues among the nursing team.

## Introduction

Endoprosthetic joint replacement is a life-enhancing procedure for millions of people around the world, which provides pain relief, restores function and preserves independence, especially in elderly patients. In Germany, primary total knee or hip arthroplasty is among the most common procedures, with an increase in the number of surgeries of up to 45% predicted for the year 2040 [1]. However, periprosthetic joint infection (PJI) is a dreaded complication in orthopaedics and trauma surgery with an incidence of 24/100,000 inhabitants in Germany [2]. For patients, PJI represents a high burden and severe limitations are often unavoidable despite modern and interdisciplinary treatment concepts. The treatment is complex depending on diverse factors such as duration of infection, underlying pathogens, and condition of the implanted prosthesis and soft tissues. Options include surgical debridement with implant retention and antibiotic therapy (DAIR), one-stage revision, or two-stage prosthesis replacement in which a new revision endoprosthesis is placed after an implant-free interval. In a significant number of patients, reimplantation is not considered feasible, leaving only arthrodesis, a Girdlestone situation, or amputation as treatment alternatives. Consequences are long hospital stays with multiple surgeries, including removal of the implant, immobility, administration of local and systemic antibiotics, often associated with side effects, and further socioeconomic issues. In addition, PJI is potentially life-threatening, with increased mortality rates compared with patients who do not develop an infection after joint replacement [3, 4]. Hence, PJI has profound effects on the patients’ quality of life leading to high levels of psychological distress [5–7]. In recent years, beside the use of patient-reported outcome measures, quantitative analyses capturing patients’ experiences following one- and two stage revision for PJI were brought into the focus of orthopaedic and trauma surgery research [8, 9]. Also, the negative emotional impact of PJI on orthopaedic surgeons and the challenge of making treatment decisions was elicited by conducting in-depth qualitative research interviews [10–12]. However, for an ideal management approach, it is important to understand the experiences of all persons involved in the treatment and the impact of managing PJI on nursing staff has not been investigated yet.

As such analysis would be beneficial in terms of identifying improvable factors, this study aims at answering the following research questions utilizing a qualitative approach: (1) Is there an emotional impact of caring for PJI patients perceived by nurses? (2) What are perceived main challenges in the management of PJI?

## Material and methods

The study took place in a German university orthopedic trauma center level I.The study population was identified using a purposive sampling strategy to ensure that participating nurses had experience in the management of PJI of at least one year [13, 14]. Participants were approached face-to-face and asked for their study participation after explaining the background and the purpose of the interviews. In total, n = 22 nurses were approached, wereof n = 2 (9.1%) declined to participate. Purpose sampling was done until the data reached theoretical saturation based on data replication or recurrence in the interview transcripts, meaning that no new themes could be identified [15]. Informed consent was obtained from all participants prior to the interviews. The study was approved by the institutional ethics committee of the University Hospital Regensburg according to the Helsinki Convention (file number 21–2464-101).

A semi-structured interview guide was conceptualized by the authors developed iteratively through multiple rounds of discussion and pilot tested. In-depth qualitative research interviews were conducted in person between March 2021 and June 2021 by a female researcher with a background in psychosomatic medicine and experience in qualitative research in an undisturbed office room. The interviewer was occupied at the same hospital, however no relationship with the participant was established prior to the study commencement. Interviews lasted between 20 and 38 min. No repeat interviews were carried out and no field notes were made during the interview. The following areas were covered in the interviews: professional experience, facilitating factors, challenges and strength in the PJI management, emotional impact and support needs. No participant dropped out. In total, 20 nurses were willing to participate. Purpose sampling was done until the data reached theoretical saturation based on data replication or recurrence in the content, meaning that no new themes could be identified [15]. All interviews were audio recorded, anonymized, transcribed and translated from German to English. Transcripts were returned to all included participants for comments. Here, no further corrections were made. To ensure trustworthiness of this research [16], credibility (internal validy) was enhanced by iterative questioning and rapport building. All participants were aware, that given statements will not be assigned to them personally and will have no effect regarding their professional position. Further, confirmability (objectivity) and reflexivity was given as the interviewer had no prior relationship with the participant and no personal or professional association with the inpatient ward. To enhance the confirmability, two researchers independently analyzed the data, which was then discussed by the whole research team (n = 7).

Data were analyzed with a content analysis. During the process, first, “meaning units” were extracted from the transcripts. This were then condensed by shorting the text while preserving the essential part. Next, an abstraction took place meaning that the condensed meaning units were labelled by a code by two researchers. Then, a descriptive grouping of related content into categories took place as described previously [10]. Finally, themes were assigned to the categories, i.e., an interpretative meaning of related data (Table 1). [17]. Codes were checked for consistency by the whole research team continously returning to the interviews and meaning units and themes were discussed until consensus was reached.

**Table 1:** Example of content analysis

Meaning unit	Condensed meaning unit	Code	Category	Theme
“Often I am a bit worried about whether everything is going well for the patients, that it does not worsen and that one does not overlook anything. That they are well cared for.” P19	Feeling uneasy	Responsibility and worries	Negative feelings associated with the management of PJI	Emotional impact
It is very burdening for the patients, especially when they stay long because they get IV antibiotics. We try our best to lift them up and calm them when they are emotionally unstable. Having a psychologist in the team for these issues, that would seriously facilitate the work for me. P17	Feeling not able to adequately address patients’ emotions	Recognition of mental issues	Need for psychological support	Patients’ psychological burden
“Neither the patients nor their relatives are aware of the consequences. They think that we can always fix everything and that it takes so long and that sometimes the infection is not eradicated easily is very difficult to accept for them. As a consequence, you just say again and again what he/she should pay attention to, what he/she should not do and many just do not do it. Patients are often incompliant, which makes it difficult”. P20	Noticing that patients have insufficient knowledge about the course of disease don’t adhere to advices	Recognition of unrealistic patients’expectations	Realization of the severity of PJI	Compliance problems

## Results

**Fig. 1:**
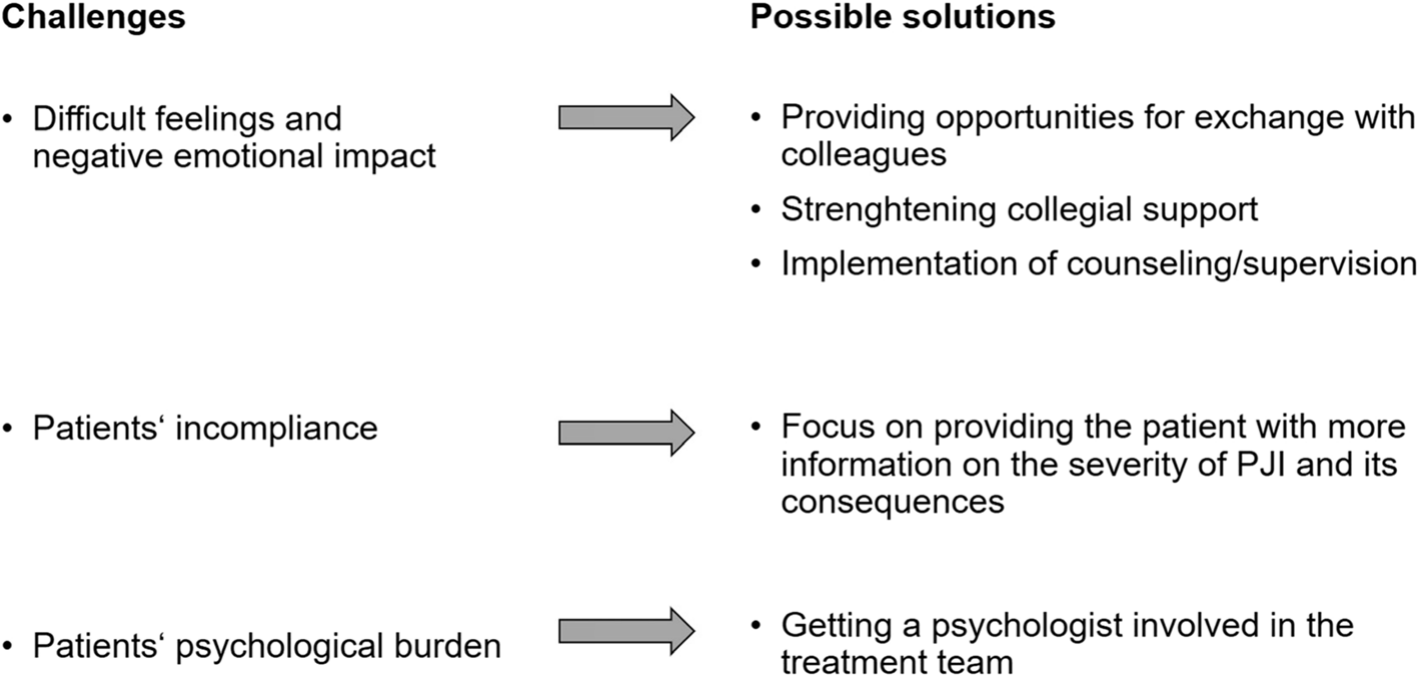
Identified challenges and possible solutions to facilitate the management of PJI

In total 20 participants with at least one year of experience working with PJI patients were interviewed (Table 2). The content analysis resulted in the identification of three main themes covering challenges in the managemant of PJI (Table 1, Fig. 1).

**Table 2:** Demographic data of the study participants with experience in managing prosthetic joint infection (PJI)

Participant	Gender	Age	Experience as a nurse	Range of experience with PJI patients (year)
P1	female	42	18	18
P2	male	36	9	5
P3	female	24	4	3
P4	female	27	6	5
P5	male	49	15	1
P6	female	55	25	1
P7	female	53	7	7
P8	male	30	11	3
P9	female	26	4	2
P10	female	24	2	1
P11	female	29	10	7
P12	female	23	4	1
P13	female	24	3	1
P14	female	21	1	1
P15	female	25	7	4
P16	female	28	4	1
P17	female	47	25	25
P18	female	27	10	5
P19	female	39	18	18
P20	female	52	27	27

### Feelings associated with the management of PJI and the need for emotional support

During the management of PJI patients, nursing staff experienced the feeling of empathy as well as disgust. “You feel for them and are empathetic, but often you also have to say that you feel a little disgusted, especially if pus or something comes out of the wound.”- P4. “Some wounds are also really disgusting, so disgust is certainly also there, and one has compassion with the patients because we know what the consequences are, and it is not always easy to deal with it”- P20. Also, participants expressed feelings of helplessness and upset. “If they have really massive pain and nothing works, and you can't help them that's really bad.”- P6. “It's very upsetting, patients are very depressed, they often don't understand why it's happening now and why it's happening to them and they try to blame someone.”—P4. Furthermore, participants experienced the feeling of overload due to the fact that time for the individual patient is limited. “You don't always go out with a good conscience. Simply, it would be required to spend more time with the patient, which unfortunately you don't have.”- P3. “We do not have the time to care for the patient the way you would actually like to”- P12. In the same stance, also worries were expressed: “Often I am a bit worried about whether everything is going well for the patients.”- P19.

Many participants reported that the negative emotional impact is reasonable and does not affect their private life significantly. “Work is work”- P9, “I always try to keep a good distance”- P5, “When I’m at home from work, I shower and that’s it”- P13. Being able to talk to colleagues about complications was considered as essential for emotional support. “We are always there for another and back us up. It makes such a difference just to talk shortly after the shift and exchange what went well and what didn’t and what is bothering you at the moment”- P10. For many, collegial support was perceived as sufficed, whereas some deemed the opportunity of professional psychological assistance as beneficial. “I think it would be helpful for some colleagues to have some sort of supervision, especially when they are new to the profession. For instance, when a patient dies, it would be important to have some support.”- P11. Participants also expressed the need to discuss their cases and the negative impact of the COVID-19 pandemic in terms of cancelled meetings. “The exchange with colleagues is crucial, but now with Corona it rarely comes to a conversation or that all sit down together”- P13. “Talking about the patients and sharing experiences is definitely a resource. We try to have case discussions as often as possible, but it is very difficult at the moment with Corona”- P18.

#### Patients’ psychological burden

All nurses recognized the patients’ burden and emphasized the mental consequences. It was pointed out that the emotional burden of PJI is high for the patients, with especially a lack of social interaction contributing to it. Not having enough time to adequately address psychological difficulties of the patients was designated as a main problem by all participants. In this stance, it was highlighted that getting a psychologist involved in the treatment team would be beneficial for all parties and significantly improve the management. “Sometimes I feel overwhelmed, because it is not possible to address the psychological burden of the patients, because the somatic symptoms are in the focus and there is not enough time to talk to the patients about it.”- P10. “I only wish to have more time available for the patient because despite changing the dressing, they also often need conversation, addressing for example the fear of amputation and so on” – P7. “Patients who are here for eternities, at one point they fall in a hole where they do not come out easily. A suggestion for improvement is definitely to strengthen the psychiatric care”- P8. “Especially patients who are in an isolation room for a long time, they crave for someone to talk to”-P2. “They certainly need more support from us in terms of having conversations, but it is just rarely possible” – P3. “It is difficult that they are so in need of a conversation and that you sometimes just don’t have the time for it” – P14. “Depression is prevalent here and I think it would be good to have psychological support, simply offer them the opportunity for having a conversation” –P17. “It is very burdening, especially when patients stay long because they get IV antibiotics. We try our best to lift them up and calm them when they are emotionally unstable. Having a psychologist in the team for these issues, that would seriously facilitate the work for me” – P17. “They would need much more support in coping with the infection. Less from the nursing side—that's simply not our job. We already talk to them, but they would need much more professional support from psychosomatics or psychology similar as the oncological patients, the patients here are somehow still not in focus, nobody is aware that they also have a psychological trauma.”—P20.

#### Realization of the severity of PJI and compliance problems

Interestingly, nearly all participants pointed out that patients do not realize the severity of PJI, which poses a major problem in terms of non-compliance. “Patients always think yes, so just a little antibiotics and that’s it” – P1. “Neither the patients nor their relatives are aware of the consequences. They think that we can always fix everything and that it takes so long and that sometimes the infection is not eradicated easily is very difficult to accept for them. As a consequence, you just say again and again what he/she should pay attention to, what he/she should not do and many just do not do it. Patients are often incompliant, which makes it difficult” – P20. “Only when the subject of amputation comes up, then I think they understand the seriousness of the situation and before that it is very underestimated” –P7. “So, they don't realize it, because as a layman you perceive an infection more like a cold, but that there are several surgeries involved or maybe a total endoprosthesis needs to be removed and debrided, which is more time-consuming, that is underestimated”- P9. “The biggest challenge is the adherence of the patients because they are just so intransigent. You make an effort and try to do everything and 5 min later they go to smoke or manipulate the wound, not understanding the seriousness of possible consequences”- P7. To improve these aspects participants suggested that the communication between physicians/caregivers and patients could be optimized. “A good thing would be more support from the medical side that the physicians repeat to explain the situation to the patients, often they listen to the doctors more than to the nursing staff”- P7. “I guess, it would be possible to inform the patients a little more proactively” – P13. “In my opinion every infectious patient needs a contact person, who is available to explain everything to them” –P11.

## Discussion

In this study, personal experiences, strength, challenges and factors facilitating the management of PJI were identified using a qualitative approach. Previously, similar studies were carried out revealing the negative impact of PJI on the patients as well as the surgeons [8–12]. However, the nursing staffs’ perspectives on strengths and challenges in the care of PJI patients has not been evaluated yet.

### Feelings associated with the management of PJI and the need for emotional support

For an ideal management approach, it is important to understand the distinct challenges for all persons involved in the treatment and increase the awareness for improvable aspects regarding the optimization of the daily clinical procedures. Facilitating associated psychosocial challenges is essential as patient care can be adversively affected by personal stress [17]. Accordingly, in units, which are characterized as having adequate staff, good administrative support for nursing care, and good relations between doctors and nurses, patients were more than twice likely as other patients to report high satisfaction with their care [18]. A patient-caregiver relationship requires intensive emotional involvement and many feelings reported were comparably with those previously described by physicians dealing with PJI [10, 11]. Here, collegial support and the exchange of experiences was considered as crucial for coping with difficult feelings, which is consistent with other studies identifying the quality of the relationships among the nursing team as well as with the physicians as a protective aspect for burnout symptoms [19–21]. Similarly, the importance of a multidisciplinary supportive team was also emphasized by surgeons concerned with infectious complications [11]. Whereas it was demonstrated that simple interventions such as listening to audio recorded mental exercises for daily 10 min over one months, significantly improved nurses’ relaxation, and life satisfaction [22], most of the participants expressed that assistance beyond talking to colleagues is not needed. However, some deemed the implementation of supervision as helpful, which has also been identified as a prevention strategy against burnout syndrome in nurses [23].

### Patients’ psychological burden

Not having the timely resources to address the psychological burden of the patients adequately was identified as a major problem in the management of PJI patients. It is well known, that PJI has a profound impact on the patients leading to high levels of psychological distress [6]. Besides the fact that PJI is potentially life threatening with five-year mortality rates up to 21% for PJI [24, 25], patients also have to deal with fears such as losing their independence and mobility [8]. A recent retrospective study showed that more than 30% of PJI patients reveal moderate to severe symptom burden on an ICD-10 based symptom rating even years after successful treatment [7]. Also, it was shown that the prevalence of mental health conditions was higher in patients undergoing septic revision total knee arthroplasty (TKA) compared to aseptic revision TKA and primary TKA, suggesting that a call for action for mental health support for PJI patients is required [26]. Whereas the need for psychological support has been explicitly reported by patients [9], the mental well-being of infection patients has hitherto rarely been focused in trauma surgery. So far no randomized controlled trials evaluating the effect of psychological interventions on treatment outcomes has been conducted, highlighting the lack of adequate strategies to address the mental burden of PJI [27]. Here, all participants reported that involving a psychologist in the treatment of PJI would facilitate their workload. Hence, the implementation of counseling as part of the standard care should be advocated. Resources should be allocated for necessary prevention priorities in order to improve treatment outcomes of PJI patients and enhance the working conditions of nurses.

### Realization of the severity of PJI and compliance problems

Additionally, noncompliance of patients was identified as a main challenge in the management of PJI. The majority of participants pointed out, that PJI patients do not realize the severity and that imparting knowledge on the consequences of the infection can be improved. Previous research has indicated that patients’ understanding of their condition and treatment is positively correlated with adherence and theoretical frameworks to implement compliance-enhancing strategies were developed [28, 29]. Patient adherence is a complex issue and the treatment team should be familiarized with providing effective patient education [30]. The nurses’ perspective that patients’ expectations on the course of the disease do not match the reality is also in line with other studies reporting that the majority of patients overestimates the benefit of medical interventions [31]. Especially, patients preparing for total joint replacement were shown to have high expectations for pain relief and the ability to walk [32]. In the case of PJI, patients wish for a only one surgery, a short time to return to normal activites, and no side effects from antibiotics [33]. Hereby, the fulfilment of patients’ expectation is positively correlated with significantly increased health-related quality of life and patients’ satisfaction [32, 34]. Thus, in turn, the patients’ expectations on the course of disease not matching the reality as reported by the nurses may be a contributing factor to the psychological issues associated with PJI.

A limitation of this study is that only experiences of nursing staff in one hospital specialized on bone and joint infection were captured. Thus, the generalizability of the results may be limited, and findings may slightly differ when transferred to centers with less expertise in the management of PJI as well as to the healthcare in other countries. Also, the study cohort was predominantly female, however, no gender-specific differences, especially regarding the reported emotional impact of PJI were noticed. In addition, the time of the interviews was relatively short (twenty to thirty-eight minutes). However, at the end each participant was asked whether there is anything else they would to add, or anything they wish to talk about that we have not covered already, which was denied by all participants.

In conclusion, this study evaluated the negative emotional impact of PJI on nurses. Having collegial support and opportunities for case discussion was perceived as a sufficient coping strategy. Further, the psychological burden of the patients and non-compliance due to a lack of realization of severity were identified as major challenges in the management of PJI. Therefore, involving psychologists in the treatment and implementing compliance-enhancing strategies as well as opportunities for professional counselling can be beneficial future directions and facilitating factors. Acknowledging how nurses perceive the management of PJI is an important information for stakeholders in the health care system and beneficial for improving clinician-nurses communication. Increasing the knowledge of challenges along with PJI treatment contributes to optimising the work environment, creating organizational structures which enhance opportunities for exchange and prevent mental health issues among the nursing team. Thus, in turn, improved professionals’ satisfaction will be beneficial for patient outcomes.

## Data Availability

The datasets generated and analysed during the current study are available. from the corresponding author on reasonable request.
